# The structure of the binary methyltransferase-SAH complex from Zika virus reveals a novel conformation for the mechanism of mRNA capping

**DOI:** 10.18632/oncotarget.23223

**Published:** 2017-12-14

**Authors:** Chatrin Chatrin, Sandeep K. Talapatra, Bruno Canard, Frank Kozielski

**Affiliations:** ^1^ Department of Pharmaceutical and Biological Chemistry, UCL School of Pharmacy, WC1N 1AX, London, United Kingdom; ^2^ CNRS, Aix Marseille University, AFMB UMR7257, Marseille, France

**Keywords:** zika virus, flavivirus, methyltransferase, methylation, mRNA capping

## Abstract

Zika virus, a flavivirus like Dengue and West Nile viruses, poses a significant risk as a pathogen in the category of emerging infectious diseases. Zika infections typically cause nonspecific, mild symptoms, but can also manifest as a neurological disorder like Guillain-Barré syndrome. Infection in pregnant women is linked to microcephaly in newborn infants. The methyltransferase domain of the non-structural protein 5 is responsible for two sequential methylations of the 5′-RNA cap. This is crucial for genome stability, efficient translation, and escape from the host immune response. Here we present the crystal structures of the Zika methyltransferase domain in complex with the methyl-donor SAM and its by-product SAH. The methyltransferase-SAH binary complex presents a new conformation of a “closed” or “obstructed” state that would restrict the binding of new RNA for capping. The combination and comparison of our new structures with recently published Zika methyltransferase structures provide a first glimpse into the structural mechanism of Zika virus mRNA capping.

## INTRODUCTION

Zika virus (ZIKV) is a member of *Flaviviridae* family, genus Flavivirus, which includes several mosquito-borne pathogens, such as Dengue virus (DENV), West Nile virus (WNV), Japanese Encephalitis Virus (JEV) and Yellow Fever virus (YFV) [1]. First identified in Uganda in 1947, the virus received little attention for decades until an explosive outbreak in Brazil in 2015 caused by an introduction from the Pacific Islands. There are two major lineages of the ZIKV: first is the Asian lineage, including Southeast Asia, the Pacific region and the Americas. Second is the African lineage, including Central, Eastern and Western Africa [1, 2]. ZIKV infections in human cause nonspecific symptoms like joint pain, mild rash, conjunctivitis, but can also manifest as a neurological disorder like Guillain-Barré syndrome [3]. Infection in pregnant women is linked to microcephaly or other brain abnormalities in newborn infants [4–6]. Due to the severity of birth defects and the size of the outbreak, the World Health Organisation (WHO) declared the virus as a Public Health Emergency of International Concern in February 2016. With no vaccine or approved medication available, there remains a pressing need to develop treatment options for ZIKV infections.

ZIKV has a ∼11 kb single-stranded positive sense RNA ((+) ssRNA), which encodes a single 3423 amino acid long polyprotein. The polyprotein is further cleaved by cellular and viral proteases to yield three structural proteins (capsid/C, membrane/prM, and envelope/E which form the virus particle and mediate viral encapsidation, host attachment and entry) and seven non-structural (NS) proteins (NS1, NS2A, NS2B, NS3, NS4A, NS4B, and NS5) which are crucial in viral genome replication, evasion from the host immune system and polyprotein processing [1, 7]. Two of these ten proteins, NS3 and NS5, have enzymatic activities: NS3 has serine protease, RNA triphosphatase (RTPase), nucleoside triphosphatase (NTPase), and helicase activities. NS5 shows RNA-dependent RNA polymerase (RdRp) and methyltransferase (MTase) activities [8]. The NS5 methyltransferase domain binds GTP and GTP analogues [9–12].

The MTase domain is highly conserved in flaviviruses and is responsible for methylation of the 5′-cap of the viral genomic RNA. This capping process involves four steps [8, 13]: (i) hydrolysis of 5′-triphosphate end of the nascent RNA into 5′-diphosphate by RTPase, (ii) transfer of GMP from GTP to the 5′-diphosphate by RNA guanylyltransferase (GTase), (iii) methylation of N-7 of guanine by N-7 MTase yielding ^m7^GpppA-RNA, known as cap-0 and (iv) further methylation of the ribose 2′-OH position of the first nucleotide of RNA by 2′O MTase, yielding ^m7^GpppA^m^2′O-RNA, also known as the cap-1 structure. In flaviviruses, the NS5 MTase domain performs both N-7 and 2′ -O methylations, using *S*-adenosyl-l-methionine (SAM) as a methyl donor and generating two *S*-adenosyl-l-homocysteine (SAH) molecules as reaction by-products. A model dubbed the ‘repositioning model’ was proposed to describe how flavivirus MTase could perform these two methylations: either (i) both methylations occur on one MTase molecule, sharing the same cofactor-binding site and the RNA substrate must be repositioned for the next methylation to take place [13, 14], or (ii) methylations involve two MTase molecules, where ^m7^GpppA-RNA needs to be dissociated and binds to the second MTase molecule for the next methylation to take place. Conceptually, both models imply a series of distinct structural conformations along the two methylation reactions which should be blatantly different in terms of chemistry and catalytic mechanism at the methyl acceptor site, *ie.*, one N7-aromatic nitrogen and one 2′-ribose oxygen. Currently, details of a mechanism for 2′-O methylation are beginning to emerge. However, those of the N7-MTase together with intermediates of loading/discarding SAM/SAH co-substrates are still elusive. The 2′-O methylation can occur on short synthetic capped RNA oligonucleotides, whereas the N7-MTase requires longer authentic sequences [14]. Structural determinants promoting the switch between these two activities expressed in the MTase domain are still elusive.

RNAs capped by viral MTases cannot be distinguished from host mRNAs and as a consequence, both are translated into proteins by host ribosomes. Uncapped cytoplasmic 5′-triphosphate RNAs are regarded as non-self and trigger a host immune response [15–17]. The retinoic acid-inducible gene (RIG-1)-like receptors (RLRs) recognize uncapped 5′-triphosphate RNA or RNA with an incomplete cap and trigger a host immune response cascade [18]. Inhibiting the ZIKV MTase may suppress viral propagation and hence serve as a potential antiviral drug target [19, 20]. The first structures of ZIKV MTase and MTase-inhibitor complexes have recently been published and initial biochemical experiments characterizing the MTase’s activity have been conducted [21–23].

In this paper, we present two novel crystal structures of ZIKV MTase with bound SAM and SAH, which deepen our structural understanding of viral RNA capping. The combination of our structures with other recently determined ZIKV MTase structures in distinct conformational states provides additional insight into structural and mechanistic intermediates along the sophisticated flavivirus RNA capping pathway. This should extend our understanding of the target and aid future inhibitor design.

## RESULTS

### Protein purification and determination of the oligomeric state of ZIKV MTase

**Figure 1:**
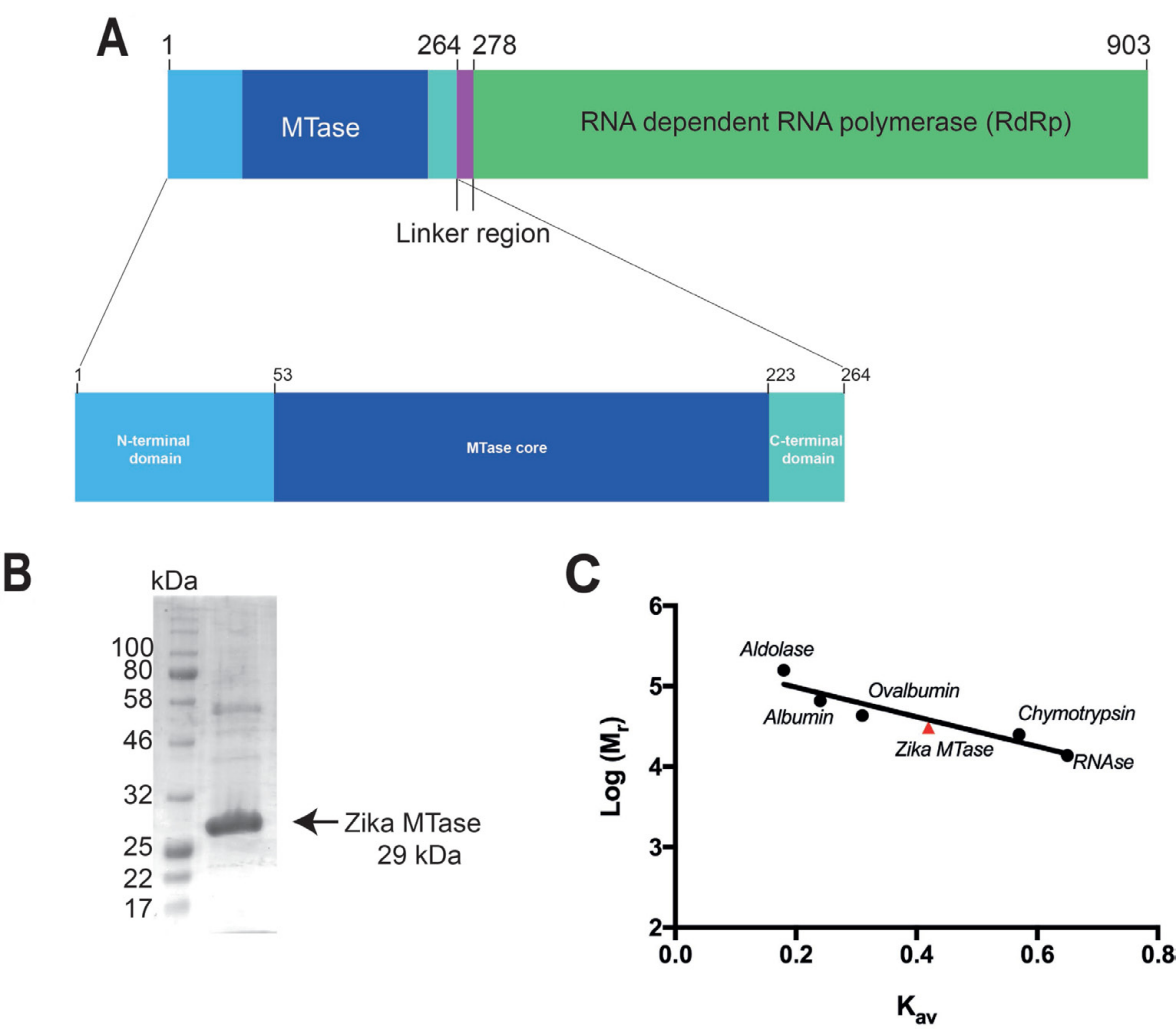
Bar diagram of ZIKV NS5 protein and purification of its MTase domain for structural studies (**A**) Bar diagram of non-structural protein 5 from ZIKV virus. The entire protein consists of 903 residues comprising an N-terminal MTase domain connected by a linker region to a C-terminal RdRp domain. The MTase domain includes the core domain (dark blue), which includes the cofactor, GTP and RNA binding pockets, and if flanked by smaller N-terminal (light blue) and C-terminal (light green) subdomains. (**B**) SDS-PAGE gel showing the purity of ZIKV MTase used for crystallization. (**C**) Determination of the oligomeric state of ZIKV MTase using gel filtration chromatography. Calibration curve for the estimation of the molecular weight where the y-axis represents the log of molecular weight of the protein standards (●) and ZIKV MTase (▲) versus their individual K_av_ calculated from gel filtration experiments.

The ZIKV MTase was 90% pure as judged by SDS-PAGE (Figure 1A and 1B). Analytical gel filtration using known molecular weight protein standards showed that the MTase behaved as a monomer with an apparent molecular weight of 30.8 kDa compared to its theoretical monomeric molecular weight of 29.4 kDa. (Figure 1C). Although ZIKV MTase-SAM and ZIKV MTase-SAH crystals were packed as tetramers and dimers respectively, both scored low interface areas of 534.4 Å^2^ and 526.7 Å^2^ as calculated by the COCOMAPS server [24]. The values are lower than the average interface areas for homodimers with monomeric molecular weight of around 30 kDa, which lies around 1800 Å^2^ [25]. This indicates the oligomers are crystallographic and not physiological.

### Brief overall description of the two structures

**Figure 2:**
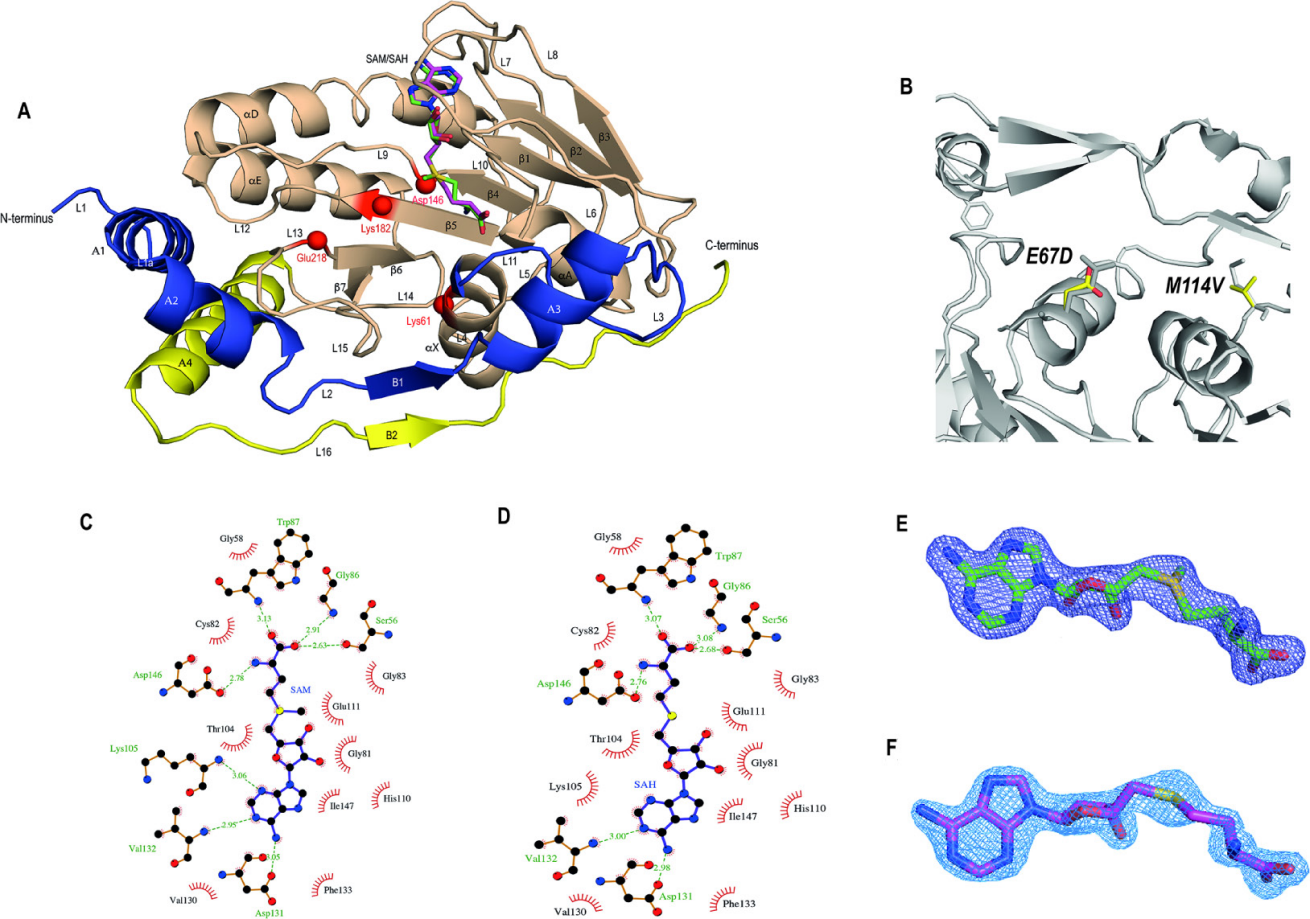
Overall description of the MTase domain with bound SAM or SAH (**A**) ZIKV MTase domain showing the overlay of the cofactors SAM and SAH shown in ball-and-stick representation. The structure consists of three subdomains: the N-terminal extension (colored in blue), the MTase core domain (shaded in wheat) containing the cofactor binding pocket and the tetrad motif (shown as red spheres) and the C-terminal subdomain (colored in yellow). (**B**) Mutations E67D and M114V in our structure overlaid with previously published structures show that they do not cause any conformational changes. Ligplot [32] presentation of the (**C**) SAM-binding and (**D**) SAH-binding pockets. Dashed green lines indicate hydrogen bonds, and the half-moon represents hydrophobic interactions between residues of the protein and the cofactor or byproduct, respectively. The electron-density of the cofactors F_o_–F_c_ difference electron density (contoured at 3σ) for (**E**) SAM and (**F**) SAH is shown as a blue mesh.

ZIKV MTase is composed of three sub-domains (Figure 2A): the major MTase core (residues 54–223) contains a Rossmann fold with seven, mixed, β-strands (β1 to β7) surrounded by two sets of α-helices (αA, αX and αD, αE) on each side. The MTase core provides the binding pocket for the methyl donor SAM and after the methylation step, it houses the by-product SAH. It also contains the catalytic tetrad motif Lys61-Asp146-Lys182-Glu218 shown to be involved in 2′O methylations to generate the cap-1 structure [26]. The small consensus motif 1 Gly-x-Gly-x-Gly-x, in our case GC-GR-GG (residues 81 to 86) is considered to be the hallmark of the SAM-binding site in the Rossmann fold for SAM-dependent methyltransferases [12, 27, 28]. The MTase core sub-domain is flanked by two smaller sub-domains termed the N-terminal and C-terminal extensions. Both are located on the same side of the core sub-domain and provide additional helical secondary elements (A1 to A4). Although all published ZIKV MTase structures originate from Asian ZIKV genotypes [2], sequences for 5KQR, 5KQS and 5M5B were from French Polynesia [21] and 5GP1 and 5GOZ were isolated from Hangzhou, China [29]. Our MTase sequence is based on an isolate from Guangdong, China and is mutated in two positions, E67D and M114V (Figure 2B). E67D is located at the end of helix αX and is solvent-exposed. Although helix αX is important for cofactor binding through residues Lys61, the mutated residue does not contribute to cofactor binding and faces in the opposite direction. The second mutation, M114V, is located in a predominantly hydrophobic pocket involving Val55, Leu44 and Val116 and fits snugly into the surrounding environment. This region is a part of the putative RNA binding pocket but whether or not this residue contributes to RNA binding is unknown.

### Description of ZIKV MTase in complex with SAM

Recently a series of ZIKV MTase-SAM complexes have been described [7, 21, 30, 31], therefore, we will only briefly explain our structure obtained with a new crystal form (see Methods, and Table 1). The cofactor SAM is bound in the central cleft formed of β-strands β1, β2, β4 and helices αX and αA (Figure 2A). The electron density of the F_o_-F_c_ map contoured at 3σ for SAM is shown in Figure 2E. SAM predominantly sits in the hydrophobic pocket with several notable hydrogen bond interactions. The adenine base of SAM forms H-bond interactions with the main chain oxygen of Asp131. The main chain nitrogen atoms of Lys105 and Val132 also form H-bond interactions with the nitrogen in the ring system of the adenine base. On the other hand, the methionine of the SAM forms H-bond interactions with the side-chains of Ser56, Trp87 and Asp146, as well as with the main-chain nitrogen of Gly86. The interactions between SAM and the residues are shown as a Ligplot interaction in Figure 2C [32]. SAM has the same conformation and interactions in all four molecules of the asymmetric unit (AU) and together with the other molecules (molecule A in PDB ID 5M5B, and 5KQR; molecules A to H in 5WZ1) provides independent structural evidence of functional interactions of the cofactor with ZIKV MTase, independently of the crystal form. It is also noteworthy that in two of the molecules, residues Ala43 to Glu46 (molecule C) and Leu44 to Val48 (molecule D) are missing, an indication of intrinsic flexibility in this region of the protein, the importance of which will be discussed later.

**Table 1:** Data collection, data processing, structure determination and refinement statistics for two new crystal forms of ZIKV MTase

	Binary MTase-SAM complex	Binary MTase-SAH complex
**Data Collection**		
**Wavelength (Å)**	0.9795	0.9163
**Resolution range**	54.53–2.0 (2.072–2.0)	39.01–2.10 (2.21–2.10)
**Space group**	P6_1_	P4_3_2_1_2
**Unit cell (a, b, c, α, β, γ)**	125.9,125.9, 125.9, 90, 90, 120	67.4, 67.4, 272.6, 90, 90, 90
**Total reflections**	603 237 (84 193)	291 550 (45 016)
**Unique reflections**	76 301 (11 052)	37 913 (5 414)
**Multiplicity**	7.9 (7.6)	7.7 (8.3)
**Completeness (%)**	100.0 (100.0)	99.8 (100.00)
**Mean I/σ (I)**	15.8 (2.4)	13.4 (3.2)
**R**_**merge**_	8.2 (63)	11.1 (68)
**Half-set correlation CC (1/2)**	72.6 (26.8)	91.7 (28.9)
**Refinement**		
**Wilson B-factor (Å**^2^**)**	32.3	28.3
**Average B-factor (Å**^2^**)**		
**Overall**	41.1	31.1
**Protein**	41.1	30.6
**Ligand**	39.2	34.5
**Solvent**	41.5	36.1
**R**_**work**_**/ R**_**free**_	20.8/25.2	18.0/23.5
**R.m.s.d., bonds**	0.012	0.012
**R.m.s.d., angles**	1.46	1.25
**Ramachandran plot**		
**..Favored (%)**	97.1	97.7
**..Allowed (%)**	2.6	1.9
**..Outliers (%)**	0.3	0.4
**Bound Ligand**	SAM	SAH

Statistics for the highest-resolution shell are shown in parentheses.

### Description of ZIKV MTase in complex with SAH

Our second, new crystal form (Table 1) reveals the structure of ZIKV MTase in complex with the by-product SAH. It contains two molecules in the AU. SAH is still bound in the SAM binding pocket and therefore represents a conformation after transfer of the methyl group. (Figure 2D). The electron density of the F_o_–F_c_ map at 3σ for the by-product SAH delineates the cofactor without the methyl group, clearly visible at this resolution (Figure 2F). SAH, like SAM, predominantly sits in the hydrophobic pocket with several notable hydrogen bond interactions. Overall, SAM and SAH show the same interactions with protein residues of the binding pocket as seen earlier for methyltransferases from other flaviviruses [33]. The adenine base of SAH forms H-bond interactions with Lys105, Asp131 and Val132. H-bond interactions with side-chains Ser56, Trp87, Asp146 and Gly86 with the SAH methionine tail are similar to those observed in SAM. In addition, the two molecules in the AU have identical interactions in the SAH binding region and there are no significant variations in the binding of the by-product, although minor changes occur due to differences in the quality of the map between protein chains. However, there are major structural differences between these two molecules in the AU, the functional importance of which will be discussed later.

### Description of the cap-binding pocket

We did not use GTP, non-methylated or methylated RNA cap analogues before or during crystallization. As expected the cap-binding pocket is empty in both of our SAM- and SAH-bound structures. Upon comparison of our structures with ZIKV MTase structures with bound GTP, ^N7Me^Gpp or ^N7Me^-Gppp there are no major differences between GTP or cap-analogue bound complexes and our “empty” cap-binding pockets. Interestingly, Phe24 (Phe25 in DENV) that is important for a stacking interaction with the guanine moiety of the cap 0 RNA still has the same orientation in our structure (Supplementary Figure 2).

### Evidence for structural changes in the putative RNA binding pocket of the SAH-bound structure

**Figure 3:**
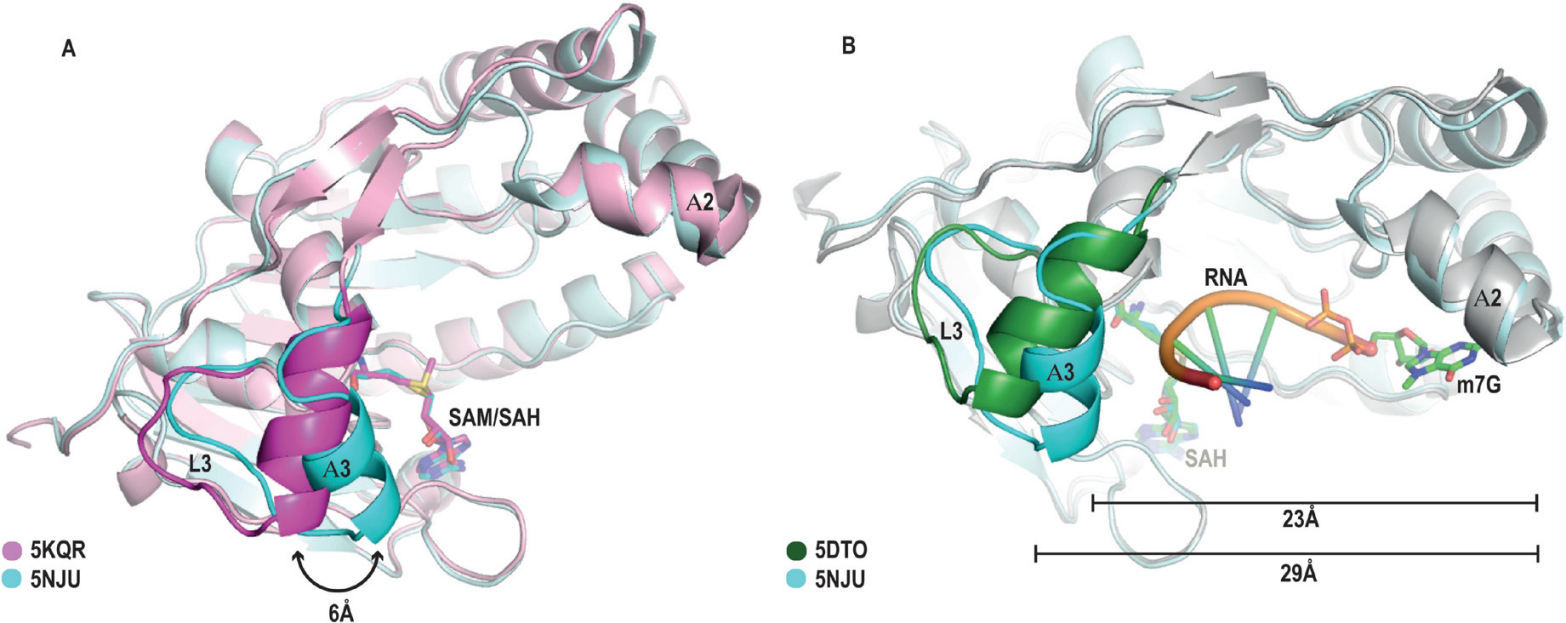
Identification of a “closed” conformation of the RNA binding cleft in the SAH-bound MTase structure (**A**) Overlay of chain A of our SAH-bound structure (cyan) with a SAM-bound structure (PDB ID: 5KQR, magenta) depicting the major conformational change in the loop L3 / helix A3 region. (**B**) Overlay of our “closed” conformation structure (L3 and A3 are coloured in cyan) with the MTase-^m7^GpppG-RNA binary complex (respective regions shaded in green) of the DENV serotype 3 structure (PDB ID: 5DTO [36]), showing the “open” and “closed” conformation of the RNA binding groove. Please note that the “closed” conformation would significantly restrict the space available for the RNA.

Although the two molecules in the AU both represent the MTase-SAH binary complex, they display significant structural changes outside the cofactor and cap-binding pockets, close to the putative RNA binding region. The short loop L3 and helix A3 are considered to be part of the putative RNA binding pocket. In chain B, spanning residues Glu38 to Gly52 (Supplementary Figure 1A, 1C), there is no electron density in this region and this part of the model is entirely absent, indicating its intrinsic flexibility. Surprisingly, in molecule A of our SAH bound structure we observed that this region is present, albeit in an entirely novel conformation, which we term the “closed” conformation (Figure 3A, 3B).

## DISCUSSION

Several viral pathogens of the flavivirus family such as DENV and ZIKV are the causing agents of a variety of diseases. Non-structural protein 5 with its two major protein domains, the RdRp and the MTase domains, is of particular interest. Their functions are essential for viral propagation and dissecting their detailed molecular mechanism is not only interesting from a fundamental point of view, but may also help to ultimately develop compounds targeting NS5. Revealing novel conformations in both domains may ultimately lead to the identification of novel inhibitor binding pockets, in particular for conformational states, which have not been revealed yet. Both RdRp and MTase domains are targets for small molecule development in both DENV and ZIKV and small molecule and fragment hits are now appearing in the literature, often from DENV or HCV-derived inhibitors, for both RdRp and MTase domains [19, 20, 22, 23, 34, 35].

### The ZIKV MTase-SAH complex is in a “closed” RNA-obstructive conformation

To assess the potential functional significance of our new conformation observed in the MTase-SAH complex we superimposed and compared it with the MTase-SAM complex (PDB ID 5KQR) (Figure 3A). We identified a significant conformational change in the loop L3 / helix A3 region, a region which is well known from previous structures and our MTase-SAM complex to display intrinsic flexibility. Whereas in the MTase-SAM complex this region is in an “open” or “permissive” conformation (Figure 3A, magenta colour), in our new MTase-SAH complex the onset of the A3 region has moved by about 6 Å towards the putative RNA binding region and is now in the “closed” or “obstructive” position (Figure 3A, cyan colour). In this “closed” orientation, A3 is one turn shorter than A3 in the “open” position. We can rule out a crystallographic artefact, as there are no residues of symmetry related molecules interacting with this region, which would impose such a conformation. The functional relevance becomes more obvious when we overlay our new MTase-SAH structure with the ternary DENV MTase complex in complex with SAH and ^m7^Gppp-RNA ([36] (PDB ID: 5DTO). ZIKV and DENV serotype 3 share 63.4% sequence identity in the MTase domain (12.3% strong similarity; 8.6% weak similarity; 15.7% different). The ZIKV MTase sequence only displays two minor amino acids insertions in loop regions compared to DENV serotype 3 MTase. The overlay between ZIKV and DENV MTase-SAM complex structures display an r.m.s. deviation of 0.5, indicating the high sequence and structural similarity between these two proteins. On superimposition, the loop L3 / helix A3 region of the DENV MTase-SAH-cap 0 RNA complex is clearly in the “permissive” orientation, with the ^m7^GPP-RNA occupying the RNA binding region between the gatekeeper region and helix A2 (Figure 3B, green colour). On the contrary, in our structure, the loop L3 / helix A3 region has moved towards the putative RNA binding region and residues of this region would clash with the RNA. It should be noted that in all other ZIKV structures and all other flavivirus MTase-SAH complexes (Supplementary Table 1 of the supplementary material section) presently available, the loop L3 / helix A3 region is either flexible or in the “open” position. We hypothesize that our ZIKV MTase structure represents a novel conformation, in which, after the RNA cap has been methylated, the by-product SAH is still bound in the cofactor-binding pocket, but the A3 region inhibits further binding of a new mRNA by being in an “obstructive” orientation. We call this region the gatekeeper, because it controls binding of new mRNA to MTase.

### A structural model for the mechanism of mRNA capping in ZIKV virus

**Figure 4:**
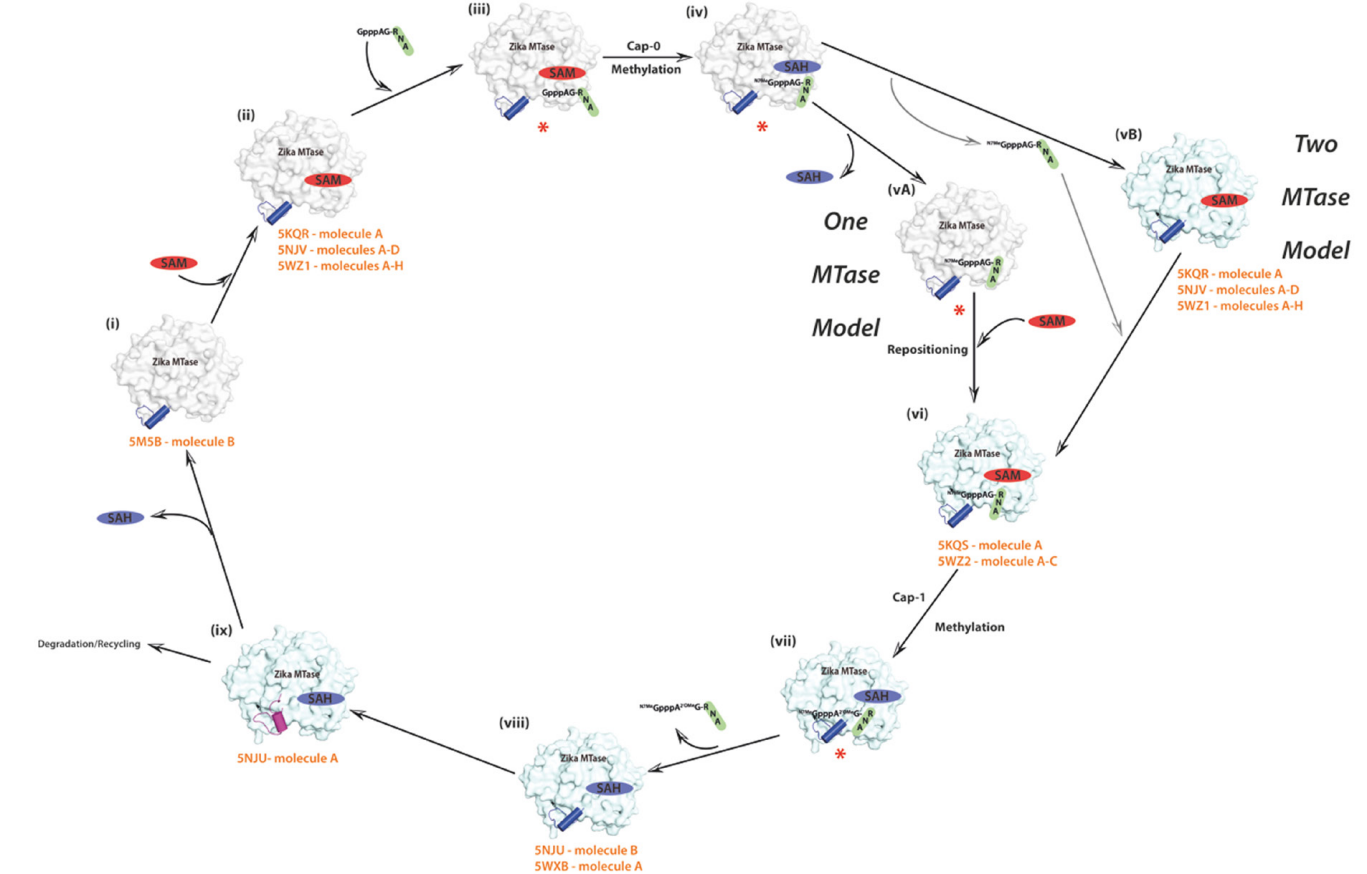
Proposed model of ZIKV MTase structure and function The 5′ end of flavivirus RNA such as ZIKV is modified by the addition of a cap structure. This cap is subsequently methylated at the N7 position of the guanosine (cap-0) and at the 2′-hydroxyl group of the following nucleotide (cap-1). Both methylations are catalyzed by the same enzyme, MTase. Numbers (i) to (viii) represent the individual steps of the capping mechanism either by the one MTase molecule or by the two MTase model. (ix) represents the new RNA obstructive stage revealed by our novel closed conformation SAH-bound structure. The surface representation of the MTase domain is shown in light grey in the one MTase model and changes to light blue in the two MTase model. The cofactors SAM and its by-product SAH are coloured in red and blue, respectively. The blue-coloured tube represents the gatekeeper loop L3/helix A3 region in either its permissive or flexible, unstructured conformation, whereas its obstructive conformation is shown in purple colour. The uncapped and capped mRNA is shown in black and green. Potential conformational states for which structures have been published are indicated with their PDB IDs (orange), whereas missing conformational states are indicated by a red asterix.

Based on recently published and our new ZIKV MTase structures we can now propose an initial structural model for the mechanism of mRNA capping in Zika virus (Figure 4) in analogy to other flaviviruses such as DENV or WNV [8, 11, 14, 37]. There are some limitations to the model: our model concentrates on structural changes in the MTase domain, which is part of the larger NS5 protein that also contains the RdRp domain. Major structural changes, in particular domain movements in the full-length NS5 protein induced by different cofactors or mRNA cap analogues are not captured in our model. Generally, short cap analogues but not cap-RNA moieties are used for the generation of MTase-cap complexes, but this is a general limitation for flavivirus research. However, since all of the MTase structures have been solved at high resolution, we are able to capture subtle changes, which would not easily be detected in the lower resolution full-length NS5 structures [38, 39].

(i) The apo-state of ZIKV MTase without cofactor or bound RNA is represented by molecule B of PDB ID 5M5B. In this structure the loop L3 / helix A3 region is in the permissive orientation. (ii) SAM, the cofactor required for the first methylation step, binds to its designated binding site. This state is represented by PDB ID 5KQR, molecule A, molecules A-H of 5WZ1 and our new structure (PDB ID 5NJV) with four molecules in the AU. (iii) The viral RNA destined for N7 guanine methylation is recognized by the MTase and binds to its binding pocket. Unfortunately, no crystal structure is available for this ternary complex. It is possible that the gatekeeper region could be in the “obstructive” conformation, since the binding mode of RNA at this stage is not known. The gatekeeper precludes binding for a 2′-O methylation competent state, but we do not know if the “obstructive” conformation could actually accommodate an RNA molecule ready to be methylated at the cap-guanine N7-position. So far it is not known yet whether SAM has to bind first, before the RNA can subsequently bind or whether SAM can bind after recruitment of the RNA. (iv) Subsequently, the methyl group from SAM is transferred to the N7 of guanine of the RNA resulting in the capping of the first site and the simultaneous generation of SAH as the by-product, which contains bound SAH and the capping analogue ^7Me^GpppA. A structure of this stage is not available.

For flaviviruses, two putative models have been proposed for the second step of cap methylation, either the one MTase model, or the two MTase model [10, 40]. In the model involving a single MTase domain that performs both N-7 and 2′-O methylations, the by-product SAH is replenished with SAM. For this step to occur, SAH has to leave the binding pocket (vA), SAM as the methyl group provider for the second methylation has to bind and the mRNA has to be repositioned (vi) so that its ^7Me^G moiety binds to the GTP binding pocket and that the 2′-OH of the adenine ribose is in proximity to SAM for the second methylation step for mRNA capping [8, 13]. If the gatekeeper was closed, it has to switch to the “open” position. It is not known for any flavivirus if RNA repositioning occurs before or after binding of SAM. No crystal structure is available for the binary complex of ZIKV MTase in complex with ^7Me^GpppAG-RNA (or one of its capping analogues), but in the absence of SAM, there is the ternary complex of MTase in complex with SAM and a very short cap analogue ^7Me^Gpp, represented by PDB ID 5KQS. Subsequently, a second methyl group is donated by SAM leading to the aforementioned cap-1 structure with methylation at the 2′-O position (vii), resulting in the generation of SAH as a by-product. For this complex, a structure is available for DENV [36]. Since the methylated viral RNA cap is not distinguishable from a normal host cap structure anymore, thus avoiding recognition by the host system, the capped RNA is released from the MTase (viii) resulting in a binary MTase-SAH complex, represented by molecule B of 5NJU and molecule A of 5WXB. Finally, the gatekeeper region changes its conformation from the “permissive” to its “obstructive” conformation (ix), restricting the binding of new uncapped RNA to the MTase domain (molecule A of 5NJU). It is not yet known whether SAH is recycled to SAM and whether NS5 is recycled or degraded.

In the model involving two distinct MTase domains, the N-7 methylated cap-RNA is transferred and correctly repositioned to a second MTase domain (vB) and the subsequent methylation at the 2′-O position then follows the same mechanism as described for the one MTase model. Currently it is not known, which of the two models is correct. Radioactive enzymatic assays of ZIKV MTase in the presence of various 5′-end modified synthetic RNAs confirmed that the domain possesses N7 and 2′O methyltransferase activities, similar to other flavivirus MTases [22]. More structural, biochemical and biophysical work is needed to better understand the mechanism of capping of ZIKV mRNA but knowledge gained from other important flaviviruses such as DENV or WNV will guide future structural work for this emerging virus.

## MATERIALS AND METHODS

### Cloning, expression and purification of ZIKV MTase

The DNA insert codon-optimised for expression in *Escherichia coli* encoding the 264 amino acids of ZIKV MTase (genebank accession AMR39831.1) in pSUMO2 expression vector was ordered from GenScript. The construct had an N-terminal octahistidine affinity tag and a small ubiquitin-like modifier (SUMO) tag with a Ulp1 protease cleavage site. The plasmid was transformed into *E. coli* BL21 (DE3) Rosetta expression cells (Novagen) and grown in 6 L of culture in Terrific Broth medium at 37° C supplemented with 34 μg/ml chloramphenicol and 50 μg/ml kanamycin to A_600_ of 1–1.2 (16–18 h). The cultures were then induced for protein expression with 0.5 mM isopropyl-β-D-thiogalactopyranoside (IPTG) for 24 h at 20° C. The cells were subsequently harvested by centrifugation for 10 min at 8000 rpm, 4° C and stored at –80° C.

The purification procedure for ZIKV MTase was similar to a previously published one [21] with some modifications. Cells were lysed by sonication (16m amplitude, 4° C, 10 cycles of 30 sec ON and 60 sec OFF) after resuspending in buffer A (50 mM 2-(4-(2-Hydroxyethyl)-1-piperazinyl)-ethansulfonic acid (HEPES), pH 7.2, 500 mM NaCl, 5% glycerol and 5 mM β-Me) supplemented with 1 mM phenylmethylsulfonyl fluoride (PMSF). The lysate was then cleared by centrifugation at 20,000 rpm for 1 h at 4° C and then loaded onto a 5 mL HisTrap FF column (GE Healthcare) pre-equilibrated with buffer A. The column was then washed for 50 column volumes (CV) with buffer A supplemented with 25 mM imidazole. Finally, the bound protein was eluted on a gradient of buffer A from 25 to 250 mM imidazole for 20 CV and collected as 2 ml fractions in a 96-well block. The fractions containing the MTase protein were pooled for subsequent steps. Dialysis using SnakeSkin™ dialysis membrane with 10 kDa MWCO (molecular weight cut off) (Thermo Fisher Scientific) and cleavage of the His-SUMO tag by Ulp1 protease (ratio protease to protein of 1:100) for 16–18 h at 4° C were performed simultaneously on the pooled protein. Dialysis buffer D contained 50 mM HEPES pH 7.5, 500 mM NaCl, 5% glycerol and 5 mM β-Me. The cleaved protein was then loaded onto a 5 mL HisTrap FF column pre-equilibrated with buffer D and the unbound cleaved protein containing flow-through was collected as 2 ml fractions.

The cleaved protein was then diluted 10-fold in ion exchange buffer E (50 mM HEPES pH 7.5, 5% glycerol and 5 mM β-Me) to reduce the NaCl concentration from 500 mM to 50 mM, and loaded on a 5 mL cation exchange HiTrap SP FF column (GE Healthcare). The column was washed for 10 CV with buffer E and the protein was eluted using gradient elution of ion exchange buffer from 0 to 1 M NaCl. The protein was further purified by size exclusion chromatography using a XK 16/100 Superose 12 column (GE Healthcare) in gel filtration buffer F (50 mM HEPES pH 7.5, 400 mM NaCl 5% glycerol, 2 mM β-Me). The protein fractions were pooled and concentrated to 10 mg/mL using a Amicon^®^ Ultracel 10 MWCO concentrator. The protein was frozen in liquid nitrogen and stored at –80^° C^.

### Crystallization of ZIKV MTase

Crystallization screens were set up using various commercial screens. Crystals for ZIKV MTase appeared after 5–7 days in 0.1 M citric acid pH 5.0 and 2 M NaCl at 18° C. The crystals were further optimized by streak seeding to obtain single crystals for diffraction measurements. In addition, reproduction of the ZIKV MTase crystals from the first published condition [21] was also carried out. The crystals grew in 0.1 M Tris pH 7.0 and 21% PEG 400 at 18° C. However, crystals grew in a new crystal form. Neither SAM nor SAH were added during the purification procedure or during crystallization and therefore originate from *E. coli*.

### Analysis of the oligomeric state using gel filtration

Gel filtration analysis of the MTase domain was performed to determine the oligomeric state of the protein (XK 16/100 Superose 12 column (GE Healthcare); 50 mM HEPES pH 7.5, 400 mM NaCl 5% glycerol and 2 mM β-Me). Experiments were conducted using a flow rate of 1 ml/min and an injection volume of 1 ml. Prior to running the MTase, the column was calibrated with proteins of known molecular mass (ribonuclease A: 13.7 kDa; ovalbumin: 43 kDa; albumin: 66 kDa; aldolase: 150 kDa; ferritin: 440 kDa; Blue Dextran 2000, >2000 kDa). The K_av_ values were calculated for these calibration proteins [(V_e_ - V_o_)/(V_c_ - V_o_)], where V_e_ is the elution volume, V_o_ is the void volume and V_c_ is the column volume and the K_av_ values were then plotted against the log of the molecular weights of the standards. The molecular mass of ZIKV MTase was calculated from the resulting equation and subsequently used to determine the oligomeric state of the protein. Protein-protein interface areas for each molecule in the crystal packing were calculated using COCOMAPS/bioCOmplexes Contact MAPS [24] by uploading the coordinate files to the server.

### Data collection, structure determination and refinement

ZIKV MTase crystals grown in 0.1 M citric acid pH 5.0 and 2.0 M NaCl on a 1:1 ratio of protein and reservoir solution in hanging drops were cryo-protected (15% w/v glycerol in 1.2 fold of reservoir solution) and flash-frozen in liquid nitrogen. The reproduced crystals grown in 0.1 M Tris pH 7.0 and 21% PEG 400 were cryo-protected in 1.2 fold of reservoir solution containing 9% sucrose, 2% glucose, 8% glycerol and 8% ethylene glycol, as previously described [21].

Diffraction data for individual crystals were collected at *beamlines I04 and I04-1* at Diamond Light Source. Data were processed using either XDS [41] or iMosflm [42] and scaled to resolutions as mentioned in Table 1 [43]. The structures of ZIKV MTase were solved by molecular replacement (PHASER MR in CCP4 suite) using ZIKV MTase (PDB code 5GOZ, [29]) as a search model. All structures were initially refined with REFMAC5 [44]. Electron density and difference density maps, all σA-weighted, were inspected, and the models were improved using Coot [45]. Further refinement of the structures was performed using PHENIX [46]. The calculation of R_free_ used 5% of data. Crystallographic and refinement statistics are given in Table 1.

Crystals of the binary MTase-SAM complex contain four molecules in the AU. Molecule A covers residues Thr4 to Arg264, molecule B contains residues Thr4 to Ala265, molecule C includes residues Gly5 to Arg42 and Gly47 to Arg264, and molecule D covers residues Thr4 to Ala43 and Ala49 to Arg264. All molecules contain the cofactor SAM. Chain C and Chain D have disordered regions of Ala43 to Asp46 and Leu44 to Val48, respectively. The second crystal form containing the binary MTase-SAH complex (one SAH / molecule) contains two molecules in the AU. Whereas molecule A includes residues Gly5 to Arg264, molecule B covers residues Gly5 to Arg37 and Val53 to Arg264. The missing region Glu38 to Gly52 is known to be part of the putative RNA binding pocket.

## SUPPLEMENTARY MATERIALS FIGURES AND TABLE



## Abbreviations

ZIKV: Zika virus
DENV: Dengue virus
WNV: West Nile virus
JEV: Japanese Encephalitis Virus
YFV: Yellow Fever virus
SAM: *S*-Adenosy-l-methionine
SAH: *S*-Adenosy-l-homocysteine
AU: Asymmetric unit
GTP: Guanosine-5′-triphosphate
RNA: Ribonucleic acid
DNA: Deoxyribonucleic acid
HEPES: 2-(4-(2-Hydroxyethyl)-1-piperazinyl)-ethansulfonic acid
β-Me: β-mercaptoethanol
PMSF: phenylmethylsulfonyl fluoride
