# Supplementary material for: The structure of the binary methyltransferase-SAH complex from Zika virus reveals a novel conformation for the mechanism of mRNA capping
Source: Oncotarget. 2017 Dec 14;9(3):3160–71. doi: 10.18632/oncotarget.23223 (PMC5790454; doi:10.18632/oncotarget.23223)
Supplement: Supplementary file 1 [file oncotarget-09-3160-s001.pdf]

# The structure of the binary methyltransferase-SAH complex from Zika virus reveals a novel conformation for the mechanism of mRNA capping

## SUPPLEMENTARY MATERIALS

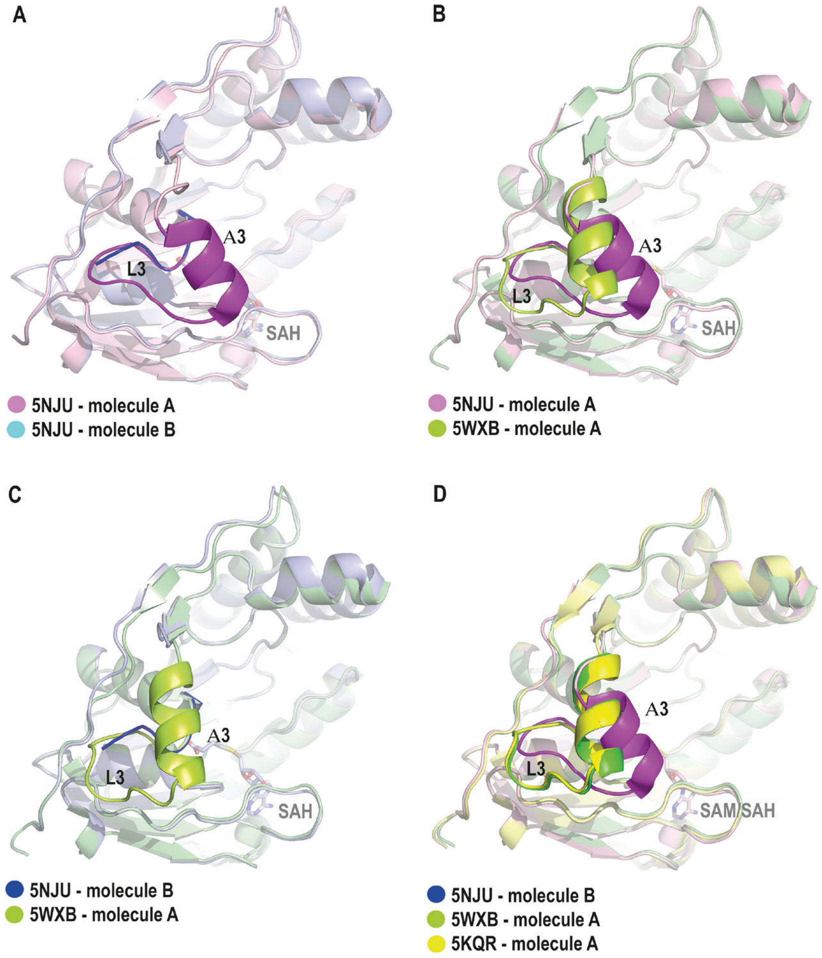


**Supplementary Figure 1: Overlay of the "closed" and "open" conformation of the RNA binding cleft in the SAH/SAM-bound MTase structures.** (**A**) Overlay of the "closed" conformation represented by chain A of our new SAH-bound structure (PDB ID: 5NJU, magenta) with the disordered region of the SAH-bound structure (PDB ID: 5NJU, pale blue). (**B**) Overlay of the "closed" conformation represented by chain A of our SAH-bound structure (PDB ID: 5NJU, magenta) with a SAH-bound structure with an "open" conformation (PDB ID: 5WXB, green) depicting the major conformational change in the loop L3/helix α3 region. (**C**) Overlay of chain B of our SAH-bound structure (PDB ID: 5NJU, pale blue) depicting the disordered region of the SAH-bound structure with chain A of the other SAH-bound structure (PDB ID: 5WXB, green). (**D**) Overlay of the "closed" conformation structure in chain A of our new SAH-bound structure (PDB ID: 5NJU, magenta) with a SAH-bound structure (PDB ID: 5WXB, green) and a MTase-SAM complex (PDB ID: 5KQR, yellow) highlighting the major conformational change in the loop L3/helix α3 regions.

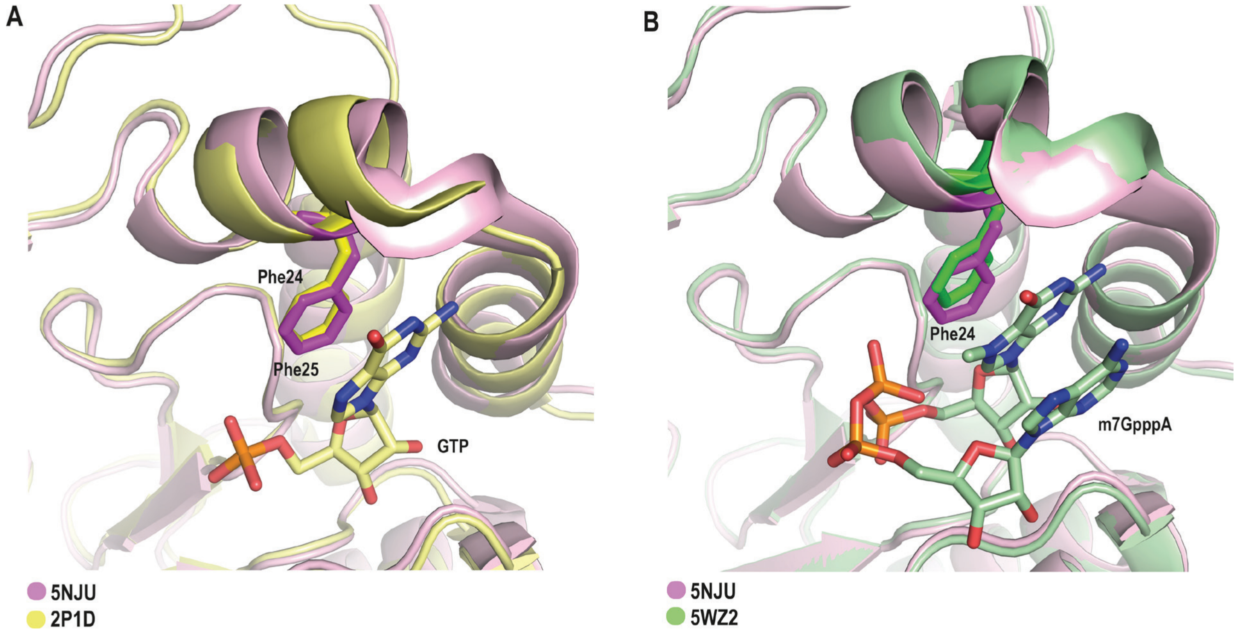


**Supplementary Figure 2: Overlay of cap binding pockets reveal no significant changes in the absence or presence of cap structures.** (**A**) Overlay of Phe24 of our SAH-bound structure (PDB ID: 5NJU, magenta) with Phe25 of the dengue MTase cap-bound structure (PDB ID: 5P1D, yellow) depicting no major conformational changes. (**B**) Overlay of Phe24 of our SAH-bound structure (PDB ID: 5NJU, magenta) with Phe24 of the Zika MTase cap-bound structure (PDB ID: 5WZ2, green) Again, no major conformational changes were observed for this key residue.

**Supplementary Table 1: Structural comparison between "open" and closed" states of MTase-SAH complexes from available flavivirus structures**

| PDB ID | Flavivirus Family Member | Molecules in AU | Conformational State of L3/A3 Region |
|---|---|---|---|
| 5NJU | ZIKV | A | Closed |
| | | B | Unstructured |
| 3EVG | DENV-2* | A | Open |
| 3P97 | DENV-3$ | A | Open |
| | | C | Unstructured |
| 2PX2 | MVEV | A | Unstructured |
| | | B | Shorter loop build |
| 2PX5 | MVEV | A | Open |
| | | B | Open |
| 3ELY | WSLV | A | Open |
| 3EVA | YFV | A | Open |
| 3EVB | YFV | A | Open |
| 2XBM | DENV | A | Open |
| | | B | Open |
| | | C | Open |
| | | D | Open |

MTase-SAH complexes with bound cap analogues were excluded from the analysis. Zika Virus: ZIKV; Dengue virus: DENV; Yellow Fever Virus: YFV; Murray Valley Encephalitis Virus: MVEV, Wesselbron Virus: WSLV; Asymmetric unit: AU; Methyltransferase: MTase. *Serotype 2; $Serotype 3.
